# A Patient with an Ileocecal MiNEN and a Synchronous Squamous Non-Small-Cell Lung Cancer: Case Report and Review of the Literature

**DOI:** 10.1155/2021/8896254

**Published:** 2021-03-31

**Authors:** Santiago Teran, Maria Camara Jurado, Juan Antonio Nuñez Sobrino

**Affiliations:** ^1^Medical Oncology Department, Hospital Universitario 12 de Octubre, Madrid, Spain; ^2^Pathology Department, Hospital Universitario 12 de Octubre, Madrid, Spain

## Abstract

Mixed neuroendocrine non-neuroendocrine neoplasms (MiNENs) are rare tumors composed of two different histological components, one of which is of a neuroendocrine origin. Given its suggested underdiagnosis and consequent low prevalence, no clear diagnostic and treatment guidelines are available, and treatment usually follows regimens similar to that of the most aggressive component. On the other hand, multiple primary tumors (MPTs) are also rare neoplastic entities that usually confer a challenge regarding treatment options, for a regimen that comprises both the primary and the synchronous/metachronous malignancy should be used. Here, we discuss the challenging diagnostic and therapeutic management of a patient with an ileocecal MiNEN that presented along with a synchronous squamous non-small-cell lung cancer (SQ-NSCLC). The patient presented with intestinal obstruction symptoms for which he underwent an emergency resection of the ileocecal MiNEN. An initial CT scan showed an additional lung mass later identified as an SQ-NSCLC after bronchoscopy biopsy analysis. Given the rapid hepatic metastatic progression, palliative platinum-based chemotherapy was initiated, with an adequate response of the local and metastatic lesions of the MiNEN, but suggested platinum resistance and progression of the pulmonary neoplasm. Second-line treatment with pembrolizumab directed for the SQ-NSCLC was initiated; however, it was stopped after immune-mediated toxicities developed. A third-line chemotherapy scheme with carboplatin/gemcitabine was initiated, but central nervous system (CNS) progression developed, with the patient dying 11 months after initial diagnosis.

## 1. Introduction

Previously called mixed adenoneuroendocrine carcinomas (MANECs), these tumors have been subjected to a new classification by the World Health Organization (WHO) classification of tumors of endocrine and digestive organs of 2017 and 2019, respectively, changing their nomenclature to mixed neuroendocrine non-neuroendocrine neoplasms (MiNENs) [1, 2]. This change was based mainly on the fact that the MANEC terminology restricted the non-neuroendocrine component to adenocarcinoma histology, and although such configuration is the most commonly found [3], it leaves aside other possible configurations both at the histopathological subtype of the non-neuroendocrine component and the degree of differentiation of the neuroendocrine component [4, 5], the latter being the parameter that often dictates the grade of malignancy, prognosis, and management to be followed [5–7]. MiNENs are extremely rare tumors with very limited scientific bibliographic data available, showing an incidence as low as 0.01/100,000 cases per year [3].

We report the case of a patient with an ileocecal MiNEN that presented along with a secondary synchronous squamous non-small-cell lung cancer.

## 2. Case Presentation

A 71-year-old heavy smoker male patient (116PY), with past medical history relevant for stage 1 COPD, presented to the emergency room with complaints of a 2-week diffuse abdominal pain, with no nausea, vomiting, GI transit disturbances, or changes in stool appearance associated. He additionally described a 13 kg weight loss over the past year, as well as a chronic nonproductive cough, which he endorsed to his smoking habit and had not worsened recently. He denied other respiratory symptoms. Physical examination was only remarkable for hypophonesis in the left upper pulmonary quadrant and acropachy in the upper extremities. A complete blood count and serum chemistry showed no abnormalities. A chest X-ray was performed, which showed a paramediastinal mass in the left upper lobe with homogeneous density and regular edges that did not seem to deviate the upper airway or compromise the great vessels in the mediastinum. The patient was admitted for further evaluation.

**Figure 1:**
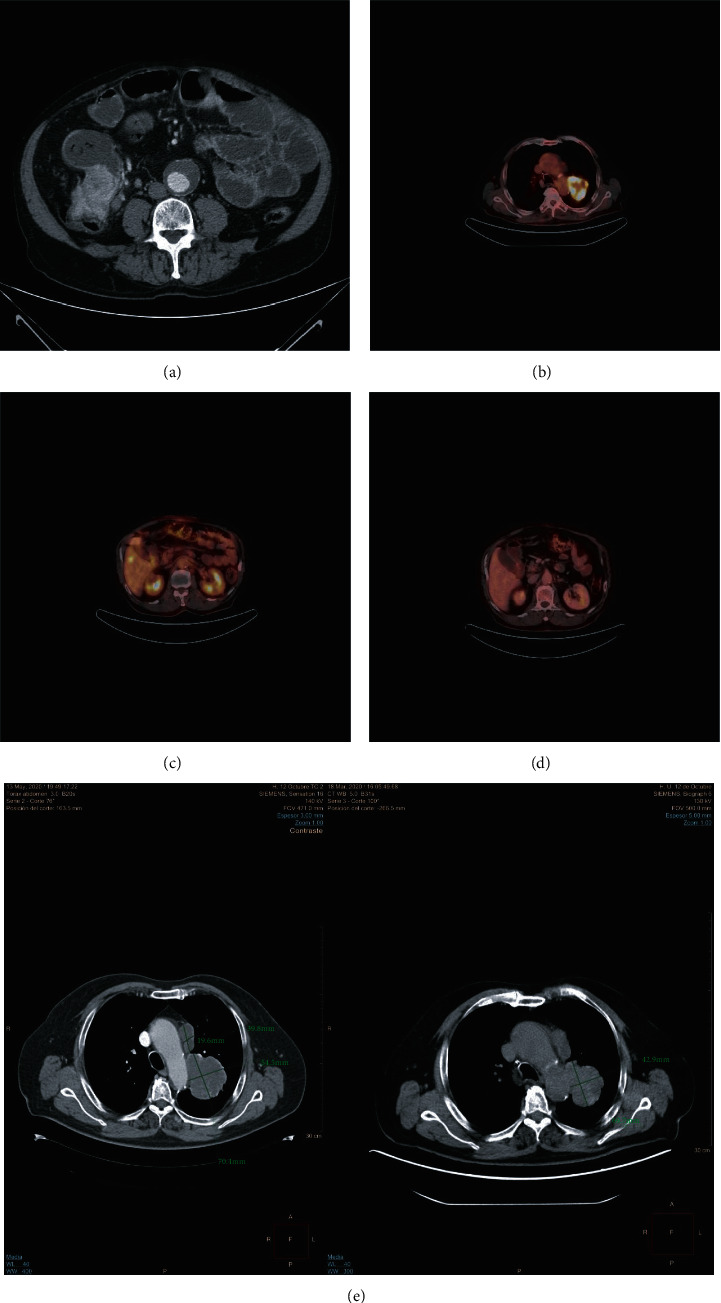
(a) Initial diagnostic CT scan shows an ileocecal mass of approx. 7 cm of diameter, with no clear radiologic signs of bowel distention or obstruction. (b) Initial PET/CT scan shows a 7 cm FDG-avid paramediastinal lesion in stretch contact with the aortic arch. (c) Initial PET/CT scan shows a metastatic lesion at the hepatic segment V. (d) Control PET/CT scan after 2 cycles of carboplatin/etoposide shows complete resolution of the hepatic lesions. (e) Control CT scan after 2 cycles of carboplatin/etoposide and 3 cycles of carboplatin/paclitaxel shows tumor progression (left image) of the paramediastinal mass, further compromising the aortic arch.

On the following days, the patient presented worsening abdominal pain, bloating, vomiting, GI transit disturbances with ultimately complete GI transit stoppage, and important abdominal distention. Physical examination showed a distended, hyperresonant abdomen, with no rebound tenderness or peritoneal irritation signs associated. A nasogastric tube was placed and an emergency abdominal CT scan was performed, which showed a 7 cm lesion located in the posterior apical segment of the left upper lobe in a paramediastinal situation, in close contact with the superior margin of the oblique fissure, main left pulmonary artery, and left margin of the aortic arch (3 cm). At the abdominal level, it showed a hypervascular lesion of neoplastic appearance of approximately 7 cm in the ileocecal junction, along with adenopathies in the ileocolic, subcarinal, mediastinal, and pulmonary hilum territories (Figure 1(a)). An emergency right hemicolectomy was performed with intraoperative findings of an ileocecal mass strongly adhered to the right parietocolic gutter, as well as several adenopathies along the ileocolonic axis, free abdominal fluid, and proximal distension of the small bowel.

**Figure 2:**
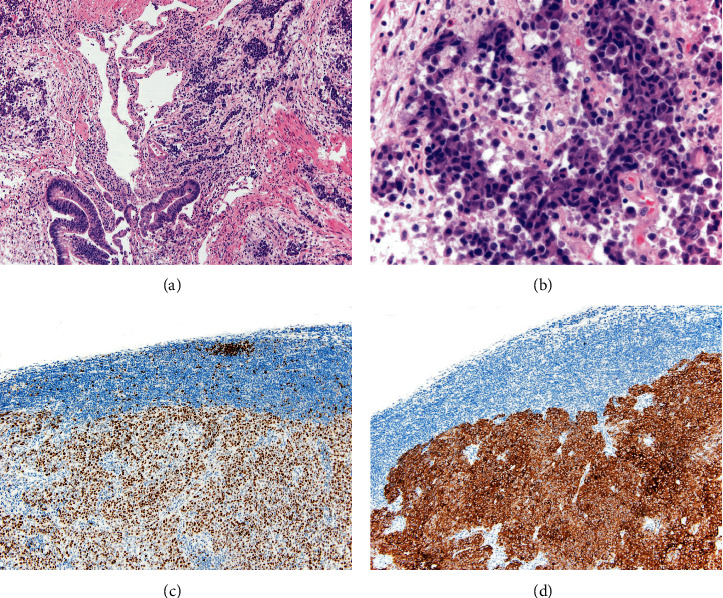
(a) Ileocolic high-grade MiNEN showing both the adenocarcinoma (mucinous glands at the bottom left) and neuroendocrine carcinoma (numerous solid irregular cords and nests, without lumen or mucin production) components. (b) High-power field of the neuroendocrine carcinoma: irregular nuclei with hyperchromasia, high nuclear/cytoplasmic ratio, no prominent nucleoli, and abundant mitotic figures. (c) Ki-67 immunostain shows a very high proliferating index (about 80%) in a NEC metastatic lymph node. (d) Strong and diffuse synaptophysin positivity in a lymph node sample, correspondent with NEC.

After adequate postsurgical evolution, a diagnostic bronchoscopy with biopsy sampling and a body PET scan were performed. The latter showed an abnormal FDG-avid activity of the left parahilar mass (6 cm × 6 cm × 4 cm) with soft tissue density, well-defined margins, and in stretch contact with the aortic arch (SUV max 24.18); newly evidenced centimetric lesions in hepatic segments IV, V, and VI (with SUVs of 6.06, 8.15, and 4.66, respectively) (Figures 1(b) and 1(c)); and para-aortic mediastinal lymphadenopathies with an SUV max of up to 16.8 for the largest one. Histopathological results of the resected colonic mass were compatible with a mixed neuroendocrine non-neuroendocrine neoplasm (MiNEN): moderately differentiated colorectal adenocarcinoma+poorly differentiated small cell neuroendocrine carcinoma (NEC) (the neuroendocrine component (NE) comprised 30-35% of the total, with high-grade characteristics and intense positivity for synaptophysin, CD56, and chromogranin A) at the ileocolic level (Figures 2(a)–2(d)), which infiltrated the muscular layer and perivisceral fat focally, also extending to the muscular layer of the appendix and its mucosa but with no serosa perforation. Lymphovascular invasion in 3/28 lymph nodes corresponded to NEC. Histopathology of the lung mass after bronchoscopy biopsies described a lesion corresponding with squamous cell carcinoma. Molecular data of the patient MiNEN showed an MSI stable marker, TMB: 11.5, as well as a PD‐L1 > 5%.

The patient was referred to medical oncology consults and began urgent palliative chemotherapy (ChT) treatment under a carboplatin+etoposide regimen, directed to the NEC component of the MiNEN given the evidence of suggested rapid progression at the hepatic level. Radiologic control after 2 cycles showed resolution of the hepatic lesions (Figure 1(d)) and no recurrence at the colonic level; however, it showed tumor progression at the pulmonary mass. To optimize the treatment for the SQ-NSCLC, without neglecting that of the digestive MiNEN, the ChT regimen was switched to carboplatin+paclitaxel, showing stability of the SQ-NSCLC after 3 cycles, but later progression with increasing size and metabolic activity at the lung mass (Figure 1(e)). Second-line monotherapy treatment with pembrolizumab directed to the SQ-NSCLC was initiated (PD-L1 greater or equal to 1%) with immune-mediated toxicities (e.g., G3 thyroiditis and cytopenias) developing, forcing to completely stop the immunotherapy treatment after only one cycle and progress to a third-line chemotherapy scheme with carboplatin+gemcitabine. Once again, tumor progression at the CNS level developed, for which he received palliative holocraneal radiotherapy treatment, along with the chemotherapy scheme proposed. The patient died 11 months after the primary diagnosis was made.

## 3. Discussion

Each component of a MiNEN tumor must constitute at least 30% of the entire neoplasm. This threshold has been arbitrarily defined based on the assumption that lower component percentages are not decisive for clinical prognosis [6]. However, it has been shown that nonpredominant components (those <30%) can be determinant given the fact that they often show aggressive histologies (e.g., neuroendocrine carcinoma and its pathological similarity with that of the small cell lung cancer (SCLC)) [4]. The most commonly found non-NE components of MiNEN are adenocarcinoma, followed by adenoma and squamous cell carcinoma [3]. On the other hand, different conformations of both components have been described at the histological level, giving us some insight into their origin. Thus, collision subtype MiNEN (two different cell types that collide and merge, but do not fuse) suggests a synchronic/metachronic origin from two different cell lineages [3, 6]. Composite and amphicrine subtype MiNENs (one cell type that displays phenotypic characteristics of others) suggest a common origin from a pluripotent stem cell that accumulates different molecular aberrations in genes such as Tp53, KRAS, BRAF, and MSI among the most relevant [3, 6, 7]. It has been suggested that the NEC component of the MiNEN originates from the adenocarcinoma one, due to the “more malignant” characteristics of the latter [8], following the adenoma-adenocarcinoma multistep sequence hypothesis [6, 9]. Nevertheless, this would contrast the fact that these tumors normally develop in neuroendocrine cell-rich organs, which would implicate neuroendocrine cells as those responsible for initiating the carcinogenic process given their stem cell potential, presence of molecular aberrations, and other physiological peculiarities, such as the lack of E-cadherin and serotonin production, characteristics that explain their metastatic and vasculogenic properties, as well as their aggressiveness and malignant potential [7, 8].

MiNEN tumors can develop at any level of the digestive tract [4], with the colon, pancreas, and biliary tract being the most commonly affected sites. The liver, on the other hand, is the most common place for metastatic involvement [3, 10]. The diagnosis of MiNEN involves imaging techniques (CT scan, MRI, US, and upper and lower endoscopy), specimen biopsy, and immunohistochemical (iHQ) markers. In the case of the latter, these are useful to define the histopathological characteristics of the tumor as well as therapeutic strategies; however, they have shown little clinical prognostic correlation [11]. Synaptophysin and chromogranin A stains are recognized as the most reliable iHQ markers for NE lineage, with the former being the one that shows greater immunoreactivity in the case of NEC [4]. On the other hand, substances produced by NE cells, such as serotonin (5-HIAA), are of little or no use in recognizing NECs, since these are generally nonsecretory unlike NETs [12]. Once the NE nature of the tumor has been identified, it is important to define the grade of histopathological differentiation (well vs. poorly differentiated), as well as the grade of proliferative criteria (G1, G2, and G3, corresponding to Ki-67: <3, Mi: <2; Ki-67: 3-20, Mi: 2-20; and Ki-67: >20, Mi: >20, respectively) [4, 6], with Ki-67 being the marker with the highest validity in case of discordant proliferative criteria [7] and the grade of histological differentiation being the most relevant parameter for discerning between NET and NEC (well-differentiated + any grade of proliferation vs. poorly differentiated + grade 3 of proliferation) [4, 6]. Finally, there are still controversy and difficulties to differentiate a NET G3 from a NEC according to the latest WHO classification, for which molecular determinations such as the expression of Rb1 and p53 have been ultimately useful to differentiate them [11].

In addition to the aforementioned, the fact that on a histopathological level the NE and non-NE components are often found in variable configurations and proportions within the tumor sample commonly leads to unrepresentative sample analysis and consequent underdiagnosis [3, 4, 10], somehow explaining their low prevalence described in different series. In line with this, within the tumor database of our center and taking into account a cohort from January 2001 to December 2019, a total of 16128 digestive system tumors were recorded (anywhere within the digestive tract). Out of these, 460 (2.85%) were classified as neuroendocrine neoplasms, of which 296 (64.3%) were neuroendocrine tumors (NETs), 158 (34.4%) neuroendocrine carcinomas (NECs), and 6 (1.3% out of NEN and 0.04% of all digestive neoplasms) were cataloged as MiNEN/MANEC. No recorded data regarding clinical outcomes of such cases was available. This data correlates to that of scientific reports showing a prevalence of 0.048% for NEN in the USA and recently increasing incidences ranging from 2.5-5/100,000 to 8.4/100,000 (in the USA and Europe, respectively), mostly thanks to earlier detection and increased use of endoscopy and other diagnostic techniques over time [11, 13]. MiNENs, on the other hand, are even rarest entities, representing 1-1.5% of all gastroenteropancreatic neoplasms [14], data that however highly surpasses that of ours. Interestingly, none of the 6 MiNEN/MANEC cases in our center was recorded before 2018. In this matter, it is important to remember that it was not until the 2010 WHO classification of neuroendocrine neoplasms that MANEC was first described as a single entity [15], which together with the underdiagnosis misleading factors mentioned before could explain our center's low MiNEN prevalence.

Despite similarities with small cell lung cancer (SCLC), NECs are less associated with smoking [16], have lower local/regional metastatic potential, and show lesser response to platinum therapies compared to SCLC [17]. On the other hand, the patient's clinical and pathological characteristics, such as the performance status, LDH levels, tumor site of origin, and Ki-67% (less response to platinum-based regimens if <55%), are all taken into account when deciding about treatment regimens, which usually follows those of SCLC [17]. Treatment of NEC differs for both the localized versus advanced (metastatic) staging of the tumor. Regarding the latter, the North American Neuroendocrine Tumor Society (NANETS) consensus suggests carboplatin/cisplatin-based first-line ChT regimens combined with etoposide/irinotecan, consistent with those of SCLC, as well as the use of gemcitabine, paclitaxel, and docetaxel, as other drug options with action both in NEC and SCLC [18]. This regimen (cisplatin+etoposide) has shown overall response rates (RR) ranging from 42% to 67% and median overall survival times (mOS) of up to 15-19 months in the first-line treatment setting [17]. In the case of second-line treatment regimens, oxaliplatin, irinotecan, and temozolomide have achieved RR of up to 33% and mOS of up to 22 months in the case of the latter [17, 19]. Curiously, these regimens have shown better outcomes in patients with Ki‐67% < 55–60%, implying the importance of a proper pathological characterization of the tumor at the time of diagnosis [17].

In the case of low, intermediate, and high-grade MiNEN, surgical excision of the primary tumor and the metastasis should always be taken into account whenever feasible [6], given the improvement in survival rates and symptom control described with these interventions [20]. Adjuvant treatment is generally directed to that of the most aggressive component [6]. Thus, in the case of low- and intermediate-grade MiNEN, regimens directed to the adenocarcinoma component are usually followed (since the NE component in these cases is usually well differentiated), while for high-grade MiNEN, regimens similar to those of poorly differentiated NEC/SCLC are followed, in consonance with recommendations for NEC treatment from the European Neuroendocrine Tumor Society (ENETS) [21]. One of the largest and most recent systematic reviews on the subject by Frizziero et al. [3] found that for localized tumors, the intention of treatment was curative with surgery in addition to perioperative ChT (60.4% surgery alone, 33.3% surgery+ChT) following in most cases regimens based on clinical guidelines for early-stage adenocarcinomas (66.7% non-NE-like regimens, versus 22.2% NEC-like regimens). On the other hand, in the advanced setting, palliative strategies followed treatment regimens for both adenocarcinoma and NEC, in similar proportions (53.3% non-NE-like regimens versus 46.7% NEC-like regimens) [3]. Other strategies such as targeting tumors with mTOR or KRAS mutations with components such as everolimus and anti-EGFRs have been proposed as possible therapeutic options, although they have not yet been implemented in a systematic manner [6, 20, 22]. Finally, despite being tumors of a NE nature, somatostatin analogs have not shown to be useful in NECs, since these lack serotonin receptor expression (SSTR-2), unlike their counterpart NETs 1 and 2 (low grade) which, given their potential to secrete serotonin metabolism products, have shown response to such treatments [6, 20]. Our case was particularly challenging given the fact that a treatment regimen that not only covered both the most aggressive component of the MiNEN and the squamous cell lung cancer (SQ-NSCLC) had to be used. In line with the aforementioned, a regimen based on carboplatin combined with etoposide and later paclitaxel was chosen. Such regimen showed an adequate response at the MiNEN level; however, tumor progression was evidenced for the SQ-NSCLC after 5 cycles and almost 6 months of treatment, and even when platinum resistance could not be certainly assumed [23, 24], second-line monotherapy with pembrolizumab directed to the SQ-NSCLC was initiated.

There are no standard second-line therapy schemes for poorly differentiated neuroendocrine carcinomas [25] and as the therapeutic option scenario broadens taking into account immunotherapy; predictive molecular/genetic biomarkers have been studied to guide clinical and therapeutic decisions [26]. Among these, MSI (microsatellite instability), which reflects a high mutational load and antigenicity with a consequent response to immunotherapy, is higher in NECs than NETs [27–29] and has been described in up to 12.4% of NEC/MANECs [25, 29, 30]. Also, a high tumor mutational burden (TMB) has been described as a positive predictive biomarker for response to PD-1/PD-L1 inhibition [26, 29, 31–34]. Several studies of immunotherapy in NEC are currently under investigation, prompted by previous positive results found in Merkel cell carcinoma, a tumor that is closely related to NEC [25]; however, mostly conflicting results from phase 1 and phase 2 trials have been encountered. Among these, a phase 1b trial (Keynote-028) which included patients with advanced solid tumors in different locations treated with pembrolizumab as monotherapy described an objective response rate (RR) of 12% for advanced PD-L1-positive carcinoid patients, as well as a 6% RR, 27% 12-month progression-free survival (PFS), and 87% overall survival (OS) for pancreatic NEC patients [26, 29, 35, 36]. This result correlates to other phase 2 studies showing RRs ranging from 5 to 17.9% [25, 37, 38]. On the contrary, some other phase 1 studies describing results of monotherapy with pembrolizumab have concluded that this was not effective in biomarker unselected populations of patients with poorly differentiated extrapulmonary NECs, like the one by Mulvey et al., describing a median PFS of 58 days, with almost half of the study population (*n* = 14) presenting early progression (PD) before the first checkup [25, 39, 40]. Another phase 2 study of pembrolizumab as monotherapy (Keynote-158) in well-differentiated NETs concluded that the PD-1 inhibitor was ineffective, with only 3% of the entire population studied (*n* = 107) presenting an objective partial radiographic response [41, 42]. Finally, similarly, other anti-PD-L1 molecules such as spartalizumab have been studied in phase 2 trials, showing once again little response (OR < 10%) in GEP NETs [26, 37, 43]. Given that our patient only received 1 cycle of pembrolizumab, it is difficult to determine any possible response to it, although the molecular profile of the MiNEN tumor with only minimally elevated TMB and MSI stable would have predicted little response to such therapy.

Regarding multiple primary tumors (MPTs), they have an incidence of 2-17%, being more common in patients with tumors or under treatments that confer long survival periods. In the case of colon and lung cancer, the incidence of second primaries accounts for 19.7% and 21%, respectively [44]. MPTs are classified as synchronous or metachronous whether the diagnosis interval between the first and second tumors is less or greater than 6 months, respectively [44]. Among the epidemiological factors related to the development of MPTs, tobacco exposure has been described as one of the most determining and commonly shared risk factors in several studies [44, 45]. Patients with MPTs have the worst prognosis compared to those with a single neoplasm, and among these, synchronous neoplasms present a statistically significant decrease in mOS when compared with the metachronous ones, given the fact that their faster development (<6 months) implies a more aggressive component of the disease [45]. On the contrary, the prognosis is also influenced by the simultaneous versus sequential diagnosis of MPTs (being worse if the diagnosis is sequential of >60 days), probably related to the fact that early simultaneous diagnosis correlates with earlier treatment initiation and better long-term outcomes [45]. Finally, MPTs imply a therapeutic challenge, since systemic therapies that cover the spectrum of both tumors, without a negative impact on the overall outcome, should be found [44].

## 4. Conclusion

We report the case of a patient with a high-grade MINEN tumor in the ileocecal region. The case is relevant since it describes the clinical, radiological, histopathological, and surgical management as well as the challenges in the treatment of this rare neoplasm. Our subject also presents a synchronous pulmonary tumor of a different lineage than the digestive MiNEN, which further complicates our patient's therapeutic options and prognosis. Finally, we also present statistical data on the incidence of MiNEN in our center to contrast with that described in the scientific literature.
